# Integrated Multi-Omic Analysis of *Mycobacterium tuberculosis* H37Ra Redefines Virulence Attributes

**DOI:** 10.3389/fmicb.2018.01314

**Published:** 2018-06-19

**Authors:** Sneha M. Pinto, Renu Verma, Jayshree Advani, Oishi Chatterjee, Arun H. Patil, Saketh Kapoor, Yashwanth Subbannayya, Remya Raja, Sheetal Gandotra, T. S. Keshava Prasad

**Affiliations:** ^1^Center for Systems Biology and Molecular Medicine, Yenepoya (Deemed to be University), Mangalore, India; ^2^Institute of Bioinformatics, International Technology Park, Bangalore, India; ^3^Manipal Academy of Higher Education, Manipal, India; ^4^School of Biotechnology, Amrita Vishwa Vidyapeetham, Kollam, India; ^5^School of Biotechnology, KIIT University, Bhubaneswar, India; ^6^CSIR-Institute of Genomics and Integrative Biology, New Delhi, India

**Keywords:** next-generation sequencing, orbitrap, genome annotation, virulence attenuation, multiomics

## Abstract

H37Ra is a virulence attenuated strain of *Mycobacterium tuberculosis* widely employed as a model to investigate virulence mechanisms. Comparative high-throughput studies have earlier correlated its avirulence to the presence of specific mutations or absence of certain proteins. However, a recent sequencing study of H37Ra, has disproved several genomic differences earlier reported to be associated with virulence. This warrants further investigations on the H37Ra proteome as well. In this study, we carried out an integrated analysis of the genome, transcriptome, and proteome of H37Ra. In addition to confirming single nucleotide variations (SNVs) and insertion-deletions that were reported earlier, our study provides novel insights into the mutation spectrum in the promoter regions of 7 genes. We also provide transcriptional and proteomic evidence for 3,900 genes representing ~80% of the total predicted gene count including 408 proteins that have not been identified previously. We identified 9 genes whose coding potential was hitherto reported to be absent in H37Ra. These include 2 putative virulence factors belonging to ESAT-6 like family of proteins. Furthermore, proteogenomic analysis enabled us to identify 63 novel proteins coding genes and correct 25 existing gene models in H37Ra genome. A majority of these were found to be conserved in the virulent strain H37Rv as well as in other mycobacterial species suggesting that the differences in the virulent and avirulent strains of *M. tuberculosis* are not entirely dependent on the expression of certain proteins or their absence but may possibly be ascertained to functional changes.

## Introduction

Among the *M. tuberculosis* strains, H37Ra, derived from *M. tuberculosis* H37 clinical isolate has been widely used as a reference strain to study virulence attenuation mechanisms (Zheng et al., 2008). Owing to its avirulent properties, it was used initially to develop tuberculosis vaccines in various animal models (Collins, 2000). Despite multiple efforts, the mechanisms of virulence attenuation are still not completely understood (Philips and Ernst, 2012). Identification of the factors involved in these processes is therefore crucial to understand the mechanisms of immune evasion and host persistence.

Several genomic differences including insertion-deletions (In-Dels) and single nucleotide variations (SNVs) have been reported in H37Ra and its virulent counterpart H37Rv using comparative genomic analysis (Brosch et al., 1999; Collins, 2000; Zheng et al., 2008). Although some of these variations have been attributed to pathogenicity and virulence (Zheng et al., 2008), the effects of most genomic differences on gene products have not yet been clearly demonstrated. While the reference genome of H37Rv has undergone several revisions to date, the genome of H37Ra has only recently been resequenced. Based on the reanalysis, almost 50% of the differences have been accredited to genome sequencing errors thereby suggesting that the genome of H37Ra is significantly more similar to H37Rv and that variations earlier attributed to virulence are no longer viable (Elghraoui et al., 2017).

At the proteome and transcriptome level, numerous studies have comprehensively analyzed changes in several *M. tuberculosis* virulent strains (Målen et al., 2011; Jena et al., 2013; Jhingan et al., 2016) including expression profiling of reference and mutant strains, identification of non-coding RNAs and mapping of transcriptional start sites to the genome of H37Rv and other clinical isolates (Bifani et al., 2000; Karim et al., 2011; Cortes et al., 2013; Periwal et al., 2015). However, studies involving H37Ra have been mostly comparative and focusing primarily on the differences in virulence and pathogenicity determining factors. Comparative proteomic analysis of membrane proteome by Målen et al. (2011), whole cell analysis by Jhingan et al. (2016) and an *in silico* approach by Jena et al. (2013) have revealed several differentially expressed proteins in H37Ra with respect to H37Rv. In addition to proteins which are distinct to each strain, they also reported proteins in H37Ra carrying mutations that may be associated with virulence attenuation. A comparative proteomic study recently published by our group (Verma et al., 2017) also revealed a distinct pattern of expression of proteins in the two strains of *M. tuberculosis*. Additionally, distinct phosphorylation-mediated changes in type VII bacterial secretion system, two-component regulatory system and fatty acid biosynthesis were observed in H37Ra compared to H37Rv. This suggests that there are significant changes in the functional proteome between the two strains despite their origin from the same parental strain. Most of these studies reported the absence of proteomic evidence for several genes in H37Ra with some being attributed to the mechanisms of virulence and pathogenicity. Regardless of the extensive literature on the subject, a detailed analysis of the expression profile of H37Ra is currently unavailable.

Using an integrated OMICs approach, we have earlier successfully demonstrated the utility of proteomic analysis in the refinement of genome annotations of several clinically important organisms (Chaerkady et al., 2011; Kelkar et al., 2011; Renuse et al., 2011; Potgieter et al., 2016; Prasad et al., 2017). In this study, we report a multi-OMICs approach for the in-depth characterization of *M. tuberculosis* H37Ra. In addition to confirming the presence of previously described proteins, our study provides both transcriptional and proteomic evidence for genes that were hitherto reported to be absent in H37Ra. We also report the identification of several novel peptides using an in-house developed proteogenomic analysis pipeline that enabled the correction of genome assembly errors and refinement of gene structures.

## Materials and methods

### *M. tuberculosis* culture

*M. tuberculosis* H37Ra strain (MTCC 300) was cultured in Middlebrook 7H9 culture medium (BD) supplemented with OADC (Oleic acid, dextrose and catalase). The tubercle bacilli culture were incubated at 37°C in a roller bottle shaker and were harvested.

### Genomic DNA extraction and whole genome sequencing

Logarithmic phase culture of *M. tuberculosis* H37Ra were harvested by centrifugation at 3,500 rpm for 5 min. The bacterial pellet was lysed in Tris-EDTA-Glucose buffer containing Lysozyme at 37°C for 2 h, followed by denaturation of proteins using SDS and proteinase K at 55°C for 40 min. Delipidation was done using cetrimide saline solution at 65°C for 10 min. DNA was extracted into the aqueous phase post-chloroform/isoamyl alcohol extraction. DNA was precipitated from the aqueous phase using isopropanol. Ethanol washed genomic DNA was resuspended in TE buffer for further use. 100 ng of genomic DNA was used for the construction of sequence library. The DNA library was prepared according to manufacturer's specifications. 100 bp paired-end sequencing was carried out using Illumina HiSeq2500 platform (Illumina Inc., San Diego, USA) (Sharma et al., 2017).

### RNA extraction and RNA-Seq analysis

0.8 OD cultures were harvested in 4M GITC solution. Bacterial pellets were resuspended in Trizol LS and 0.1 mm zirconia beads. Cells were lysed by 3 pulses of bead beating at 4,800 rpm thrice for 30 s. The cell lysates were extracted with chloroform: isoamyl alcohol and RNA was precipitated from the aqueous phase. RNA was DNase treated, followed by column purification. Construction of cDNA libraries was carried out following manufacturers' instructions. Amplified cDNA fragments were sequenced using Illumina HiSeq2500 platform (Illumina Inc., San Diego, USA) (Sreenivasamurthy et al., 2017).

### Protein extraction and sample preparation for proteomic analysis

For proteomic analysis, the bacterial pellets were lysed using 4% SDS, 50 mM TEABC, pH8.0 with heating 95°C for 20 min. The samples were then sonicated using a water bath sonicator for 60 min and centrifuged at 13,000 rpm for 10 min (Verma et al., 2017). The protein concentration in the lysate was determined using bicinchoninic acid assay (Pierce, Waltham, MA). Two different fractionation methods were employed. In the first method, 200 μg of proteins was resolved on 10% SDS-PAGE gel, and 16 gel bands were excised and destained. Proteins in the gel bands were reduced and alkylated using 5 mM dithiothreitol (DTT) at 60°C for 20 min and 20 mM iodoacetamide (IAA) at room temperature for 10 min in the dark respectively. Trypsin (modified sequencing grade; Promega, Madison, WI, USA) was added to the gel bands, and digestion was carried out at 37°C for 16 h. The peptides were extracted and subjected to desalting using C_18_ StageTips.

In-solution digestion was carried out using one mg of the protein lysate followed by HPLC-based fractionation. The protein lysate was reduced and alkylated as described earlier. The protein pellet devoid of SDS was obtained by precipitation using six volumes of ice-cold acetone at −20°C for 6 h followed by centrifugation. The protein pellet was resuspended in 50 mM TEABC, and enzymatic digestion was carried out by adding trypsin (Worthington Biochemical Corporation, Lakewood, NJ, USA) at a final concentration of 1:20 (w/w) for 16 h at 37 °C. The peptide digest was loaded onto Waters XBridge column (Waters Corporation, Milford, MA; 130 Å, 5 μm, 250 × 4.6 mm) using a manual injector attached to Hitachi HiChrom HPLC system. Fractionation of the peptide digest was carried out using a 130 min gradient, at a flow rate of 0.5 ml/min of solvent A (10 mM TEABC buffer, pH ~8.5) and solvent B (10 mM TEABC buffer, 90% acetonitrile, pH ~8.5). The gradient program was set as follows: 97% A for 20 min, 90% A for 60 min, a gradient of 35–100% of B for 25 min and further subjected to equilibration in 95% of solvent A for 25 min. The fractionated sample was finally concatenated into 24 fractions. Pooled samples were lyophilized and desalted using C_18_ StageTips.

### LC-MS/MS analysis

The peptides obtained from a total of 30 fractions were subjected to LC-MS/MS analysis using Orbitrap Fusion Tribrid mass spectrometer (Thermo Fisher Scientific, Bremen, Germany) coupled to Easy-nLC1200 nano-flow UHPLC (Thermo Scientific, Odense, Denmark). The peptide digests were reconstituted in 0.1% formic acid and loaded onto nanoViper trap column 2 cm (3 μm C18 Aq) (Thermo Fisher Scientific). Peptide separation was carried out using EASY-Spray C18 analytical column (15 cm, 75 μm PepMap C18, 2 μm C18 Aq) (Thermo Fisher Scientific) at a flow rate of 300 nL/min. A linear gradient of 5-35% solvent B (80% acetonitrile in 0.1% formic acid) over 100 min was used to resolve the peptide mixture with a total run time of 120 min including sample loading and column reconditioning. Data dependent acquisition with full scans in 400–1,600 m/z range was carried out using an Orbitrap mass analyzer at a mass resolution of 120,000 at 200 m/z. The most intense precursor ions from a survey scan were selected for MS/MS and fragmented using HCD mode with 34% normalized collision energy and detected at a mass resolution of 30,000 at 200 m/z. Peptide charge state was set to 2–6, and dynamic exclusion was set to 30 s. Internal calibration was carried out using lock mass option (m/z 445.1200025) from ambient air.

## Data analysis

### Mapping and variant detection using *M. tuberculosis* H37Ra and H37Rv reference genomes

The quality of the raw reads obtained from whole genome sequencing analysis was verified using FastQC. The filtered reads were aligned to H37Ra reference genome (NC_009525.1) (downloaded from NCBI, updated May, 2016) (Zheng et al., 2008) and NZ_CP016972.1 (downloaded from NCBI, updated March, 2017) (Elghraoui et al., 2017) using Burrows-Wheeler Alignment Tool (BWA version-0.7.15) (Li and Durbin, 2009). The alignment files were subjected to local realignment and de-duplication using the Genome Analysis Toolkit (GATK version-3.6). SNVs in the coding and promoter region were identified from each alignment file using GATK (McKenna et al., 2010). Insertion/Deletions (In-Dels) were identified from each alignment file using GATK and Pindel (version-0.2.5b8) (Ye et al., 2009). The variants and In-Dels were annotated using in-house Perl scripts. Briefly, the SNVs were annotated using reference genome fasta file and gtf file to identify if they were falling in the region of protein-coding genes. Further, these variants were categorized as synonymous and nonsynonymous SNVs with amino acid change and its corresponding position were also obtained. The In-Dels were classified into insertions and deletions with the details of protein coding genes and the number of bases inserted or deleted. All variants identified in this study were manually inspected using Integrative Genomics Viewer (IGV version- 2.3.86) (Robinson et al., 2011). The data was also compared with H37Rv reference genome to identify H37Ra specific variants with respect to H37Rv. The raw reads after quality check was aligned to *M. tuberculosis* H37Rv reference genome (NC_000962.3) using the methods described above. The SNVs and In-Dels were then compared with genetic variations reported by Zheng et al and Elghraoui et al. to identify similarities and differences in identifications.

### Transcriptome data analysis

Reads obtained from the RNA-Seq analysis were quality filtered for Phred-based base quality (Q > 20) using FastQC. The reads that passed the quality threshold were used in downstream analysis steps. BWA (version 0.7.15) with default parameters was used to align the reads against the *M. tuberculosis* H37Ra reference genome (NC_009525.1) (updated May, 2016). For the coding transcriptome, the number of reads mapped to each coding sequence (CDS) was calculated using HTseq (Anders et al., 2015) and corrected for gene length and library depth to generate normalized reads per kilobase per million mapped reads (RPKM) values (Mortazavi et al., 2008).

### Database searches for peptide and protein identification

Raw data files obtained from mass spectrometry analysis were processed using Proteome Discoverer software version 2.1 (Thermo Fisher Scientific, Bremen, Germany). The data were searched using SequestHT and Mascot search algorithms against H37Ra protein database (downloaded from NCBI, updated May, 2016) combined with common contaminants (total number of sequences: 4,143 proteins). The search parameters employed for the analysis included trypsin as the protease with a maximum of 2 missed cleavages allowed. Oxidation of methionine and protein N-terminal acetylation were set as dynamic modifications, whereas, carbamidomethylation of cysteine was specified as static modification. Precursor ion mass tolerance and fragment ion mass tolerance were set to 10 ppm and 0.05 Da respectively. Percolator node was employed to calculate False Discovery Rate (FDR). The FDR for the proteomic data in the study was < 1% at both peptide and PSM levels. The global protein FDR for the entire dataset is 4%.

### Generation of custom databases for proteogenomics analysis

For the proteogenomic analysis, four custom databases were generated including (i) six-frame translated genome database, (ii) three-frame translated RNA-Seq transcript database, (iii) alternative start codon database, and (iv) a hypothetical N-terminal peptide database. The six-frame translated genome database was created using the genome sequence of H37Ra (NCBI Reference Sequence: NC_009525.1) downloaded from the NCBI FTP site. Using in-house python scripts, six-frame translated database was created containing genomic sequences translated from stop codon to stop codon using standard (confirm) codon table. The information pertaining to the start coordinate, end coordinate and the frame of translation were listed in the header of the translated amino acid sequence. Only those translated sequences were considered which had peptide sequences with the length of ≥7 amino acids. The translated transcriptome database was created using assembled transcripts from RNAseq data generated in this study. *Mycobacterium* species are known to use GTG and TTG as alternate initiator methionine codons. Therefore, to create an alternative start codon database, the genome was translated in six frames, and wherever GTG and TTG codons were encountered, they were considered as probable translation start sites and translated as the initiator methionine in addition to valine and leucine (Kelkar et al., 2011). The hypothetical N-terminal tryptic peptide database was created by fetching all the peptide sequences that began with methionine and ended with K/R from the six-frame translated genome database. Common contaminant sequences were added to each database, and only those peptide sequences with lengths ranging from 7 to 25 amino acids were included in the databases. The search parameters employed were the same as discussed in the previous section.

### Proteogenomic analysis and workflow for manual genome annotation

The unassigned spectra obtained after searches against the reference protein database were used for proteogenomic analysis. The data were searched against the custom databases mentioned in the previous section. Peptides obtained after applying the 1% FDR cutoff criteria were selected, and genomic coordinates were obtained for these using an in-house Perl script. Briefly, the peptide sequences were mapped to the six frame translated genome fasta file and the genomic coordinates of the translated sequence was retrieved. Based on the start coordinate, end coordinate and the frame of translation, genomic coordinates for the peptides were obtained. Peptides mapping to multiple regions in the H37Ra genome and those mapping to known protein sequences were not considered for further analysis. Genome search specific peptides were categorized as follows: (1) mapping to intergenic region, (2) partially overlapping with annotated genes, and (3) completely mapping to annotated genes but translated in a different frame. Alternative gene models were predicted using two gene prediction programs—FgeneSB and GeneMark (Version 2.5) (Besemer and Borodovsky, 2005). Orthologous evidence for putative novel genes and refined gene models were obtained by sequence comparison across *Mycobacterium* species using protein BLAST (https://blast.ncbi.nlm.nih.gov/Blast.cgi). MS/MS spectra of all instances of peptides suggesting novel gene models or refinement of existing gene models were manually verified.

### Estimation of protein abundance and gene ontology analysis

The relative abundances of identified proteins in H37Ra were determined using intensity-based absolute quantitation method (iBAQ) (Schwanhäusser et al., 2011). The intensities of all the observed tryptic peptides of a given protein were summed and then divided by the number of theoretically observable tryptic peptides. Gene Ontology-based functional classification information for molecular function, biological process, and subcellular localization were fetched from Uniprot for all the proteins present in the protein database. FunRich tool was used for functional enrichment of the identified proteins in our study using the Uniprot annotation as backend database (Pathan et al., 2015).

### Data availability in public repositories

The sequencing data from whole genome sequencing and transcriptomic profiling experiment have been deposited in the SRA database (www.ncbi.nlm.nih.gov/sra). They can be accessed using identifiers SRP127305. The mass spectrometry data have been deposited to the ProteomeXchange Consortium (http://proteomecentral.proteomexchange.org) via the PRIDE partner repository with the dataset identifier PXD008555.

## Results

**Figure 1:**
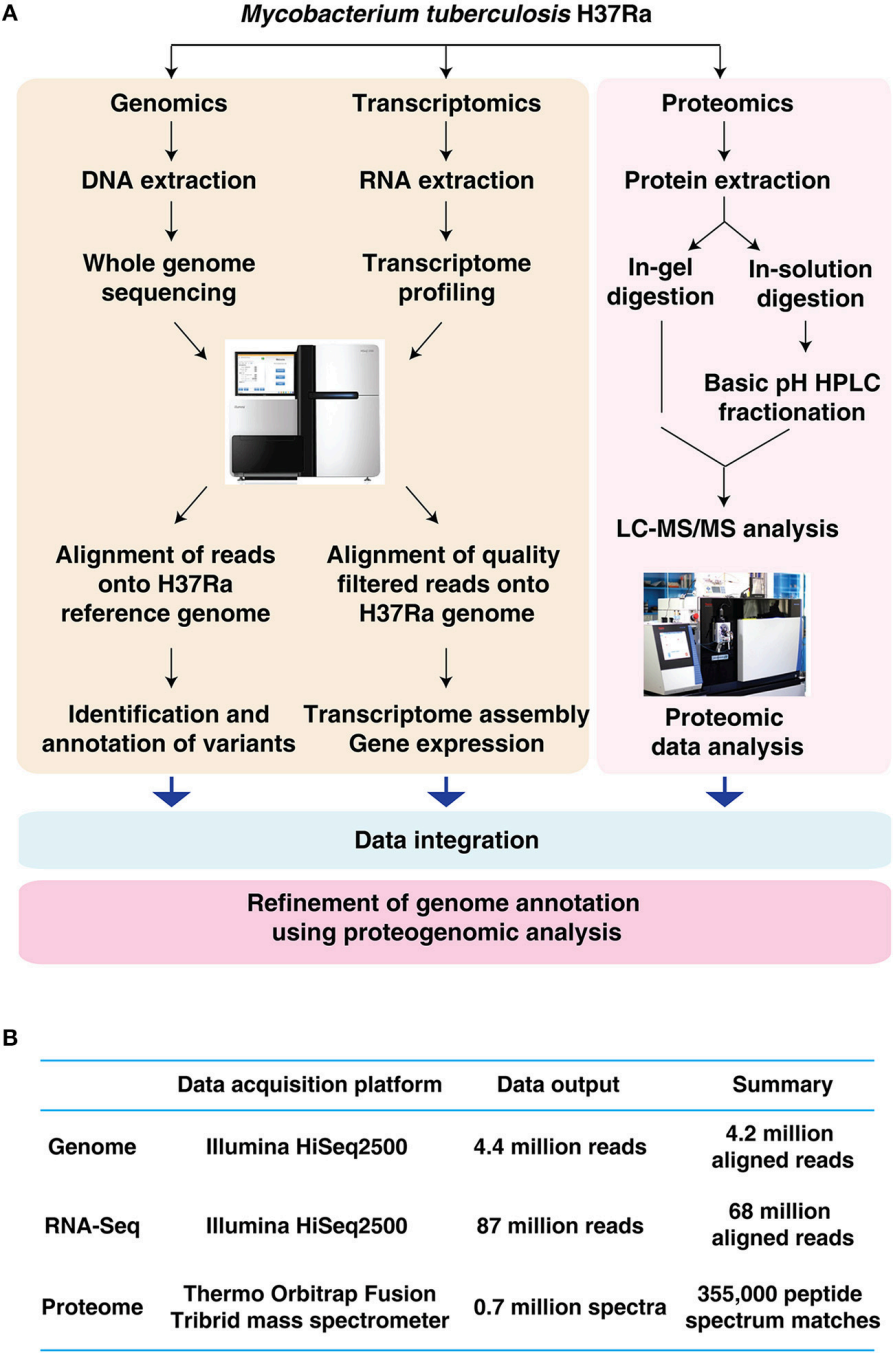
Characterization of *Mycobacterium tuberculosis* H37Ra using an integrated OMICS analysis. **(A)** Schematic representation of the workflow employed in the current study to characterize the genome repertoire of *Mycobacterium tuberculosis* H37Ra. **(B)** Summary of genomic, transcriptomic and proteomics analysis of *Mycobacterium tuberculosis* H37Ra.

In the current study, a multi-pronged OMICs approach was employed for the in-depth characterization of *M. tuberculosis* H37Ra (Figure 1A). The details of the analysis carried out in this study is summarized in Figure 1B.

### Genomic features of H37Ra

To identify the genomic features of *M. tuberculosis* H37Ra strain (MTCC300), we carried out whole genome sequencing analysis on Illumina HiSeq2500 platform. The genome of *M. tuberculosis* H37Ra comprises of 4,419,977 bp encoding a total of 4,152 protein-coding sequences (CDS). On an average, we obtained 4.4 million sequence reads at a read depth of 100X with coverage of ≥95%. The raw reads obtained from the analysis were aligned to H37Ra reference genome (NC_009525.1) and H37Ra genome (NZ_CP016972.1) analyzed by Elghraoui and group (Elghraoui et al., 2017). In all, we identified 58 SNVs, 5 insertions and 12 deletions with respect to NC_009525.1 (Supplementary Tables 1A,B) and 13 SNVs, 2 insertions and 3 deletions with respect to NZ_CP016972.1 (Supplementary Tables 1C,D).

We further compared our data with H37Rv reference genome (NC_000962.3) which resulted in the identification of 66 SNVs (Supplementary Table 2A), 16 insertions and 11 deletions (Supplementary Table 2B). We also identified mutations in the promoter regions of 7 genes (Supplementary Table 2C) including *OtsB* (Rv2006), a key enzyme in the trehalose biosynthesis pathway that is required for the synthesis of trehalose-based glycolipids including diacyltrehalose/polyacyltrehalose (DAT/PAT) and sulfolipid (SL). These mycolipids are essential constituents of mycobacterial cell wall and therefore play an important role in virulence (Kalscheuer and Koliwer-Brandl, 2014, Murphy et al., 2005). It is important to note that H37Ra fails to produce acyltrehalose which is an abundant lipid in H37Rv (Chesne-Seck et al., 2008). This lack of acyltrehalose was previously shown to be due to loss of function mutation in the PhoR transcription factor preventing transcription of the *pks2-3/4* locus. Surprisingly mutations in *pks2-3/4* do not lead to loss of virulence in H37Ra. Our data therefore suggests that mutations that affect other metabolites in the pathway may contribute to loss of virulence rather than a direct effect on acyltrehalose.

Of the total number of mutations identified in H37Ra with respect to H37Rv genome, 27 SNVs and two of each insertions and deletions were identified for the first time (Supplementary Tables 2A,B).

**Figure 2:**
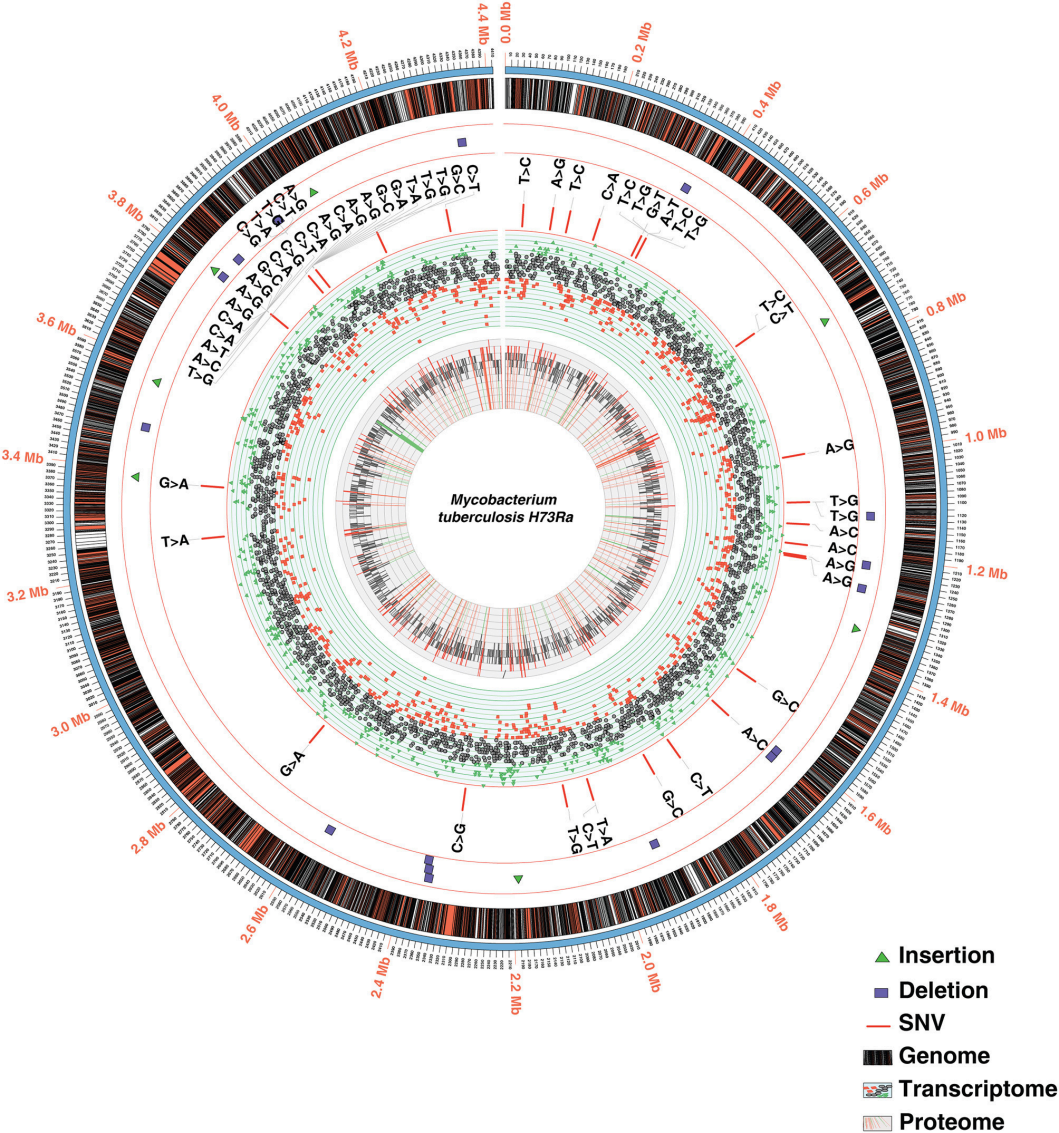
Circos plot depicting the integrated view of the data obtained through multi-OMICs approach.

In order to identify high confidence H37Ra-specific variants, we next compared SNVs and In-Dels common to all three studies. Our analysis led to the confirmation of 39 single nucleotide polymorphisms common across all the three studies including variations in *phoP* (S219L) and *espK* (I228M) (Figure 2, Supplementary Tables 3A,B). We also confirmed the stop gain mutations in phosphate ABC transporter permease -*PstA* and molybdopterin oxidoreductase. Of the earlier reported In-Dels, 14 insertions, and 9 deletions were common to all three studies validating the fact that these mutations are indeed actual changes in the avirulent H37Ra strain (Figure 2, Supplementary Table 3B). These include large deletions in *nrdH*-redoxin, IS6110 transposase and IS3 family transposase.

### Transcriptomic profile of H37Ra

Transcriptome profiling of *M. tuberculosis* H37Ra was carried out on Illumina-HiSeq-2500 sequencing platform using paired-end sequencing (100 bp paired-end reads). From the RNA-Seq analysis, 87,862,710 reads were identified resulting in 78% read alignment to the reference genome (NC_009525.1). The number of reads mapping to each coding sequence (CDS) was calculated and corrected for gene length and library depth to generate normalized reads per kilobase per million mapped reads (RPKM) values (Mortazavi et al., 2008). A total of 3,945 CDS had RPKM values of 1 or more representing 94% of the annotated genome (Figure 2, Supplementary Table 4). The RPKM values for the genes in which we identified promoter mutations were varied. The transcript level expression of *OtsB* falls in the lower 10%, whereas that of *MftE* falls in the higher 10% of the overall expression range. Additionally, transcriptomic evidence was also obtained for genes that were missed in the earlier annotation (described in Proteogenomics section).

### Proteomic landscape of *M. tuberculosis* H37Ra

In order to achieve maximum proteome coverage, we employed two fractionation techniques namely in-gel digestion and high pH HPLC-based -fractionation. The mass spectrometry-derived data were analyzed using SequestHT and Mascot search engines available through the Proteome Discoverer software suite (version 2.1). A total of 743,816 MS/MS spectra were acquired in FT-FT mode resulting in the identification of 344,436 peptide spectral matches mapping to 31,122 peptides. Overall, these peptides corresponded to 3,205 proteins, the largest report on H37Ra to date. Altogether, the identified proteins correspond to ~77.4% of H37Ra reference proteome (Figure 2). The complete lists of peptides and proteins identified in this study are provided in Supplementary Table 5A.

**Figure 3:**
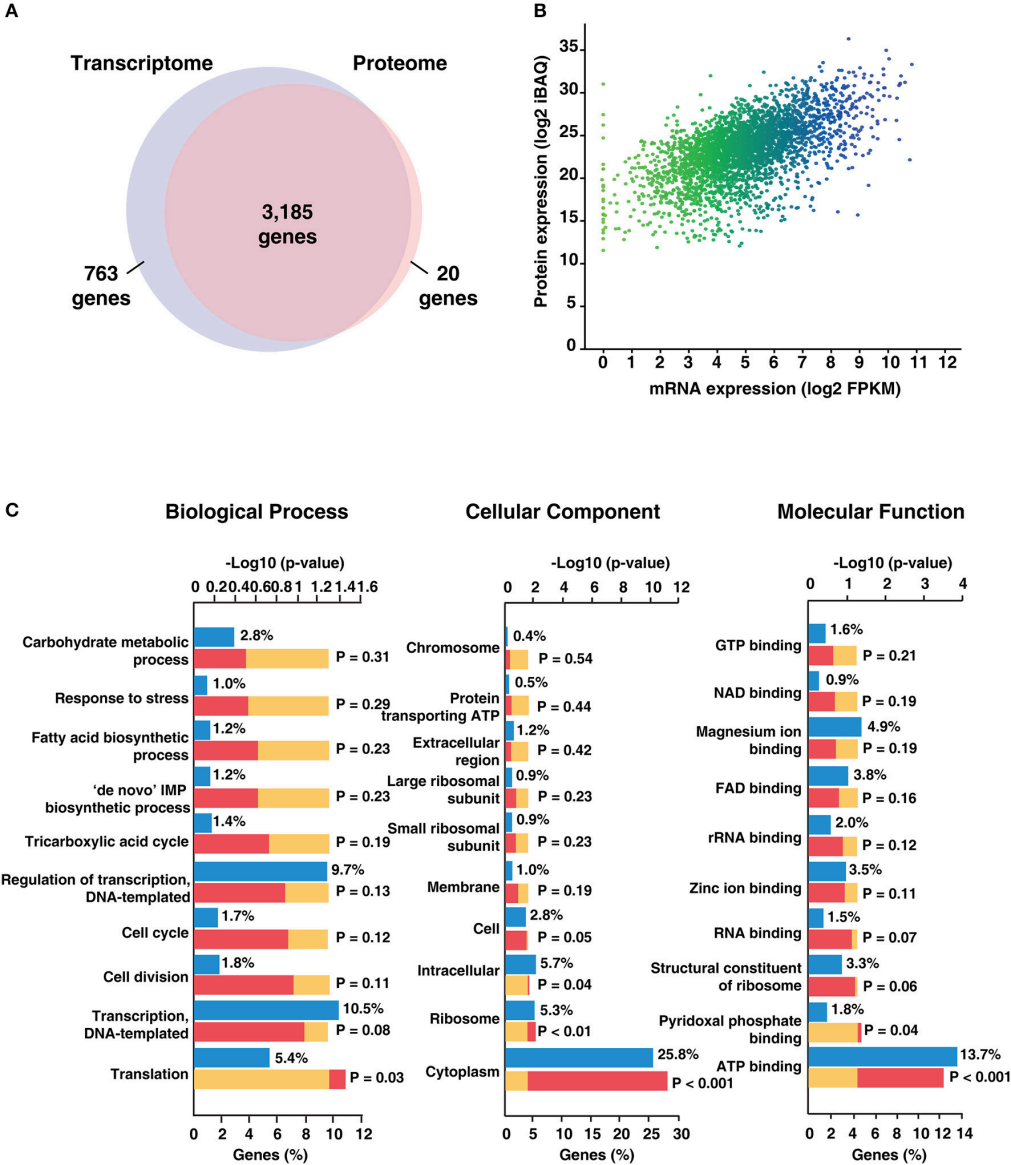
Summary of transcriptomic and proteomic analysis of *Mycobacterium tuberculosis* H37Ra.**(A)** Comparison of the mRNA and protein levels reveals ~ 80% of the genes have both transcriptome and proteome evidence. 763 genes were identified based on transcript evidence alone. **(B)** Correlation plot depicting concordance between RNAseq and proteomic data for *Mycobacterium tuberculosis* H37Ra strain. **(C)** Gene Ontology-based classification of identified proteins based on biological process, molecular function and cellular component.

To determine the extent of correlation between mRNA abundance and protein expression, we compared the proteome data of H37Ra with its transcriptome. Of the total transcripts mapped to H37Ra proteome, 80% of the transcriptome was found to have corresponding peptide evidence (Figure 3A). In addition, we identified 20 proteins exclusively through proteomic analysis and 763 transcripts exclusive from the transcriptomics data. To obtain an estimate of the protein abundance, we employed iBAQ algorithm. Spearman's correlation coefficient was computed to correlate protein and mRNA data using log-transformed iBAQ values and FPKM-based transcript abundances. A Spearman's correlation coefficient of (*r*) = 0.47) (Figure 3B) was observed corroborating with previous reports that the proteome expression does not necessarily correlate with the transcript level of expression.

### Functional annotation of the H37Ra proteome

**Figure 4:**
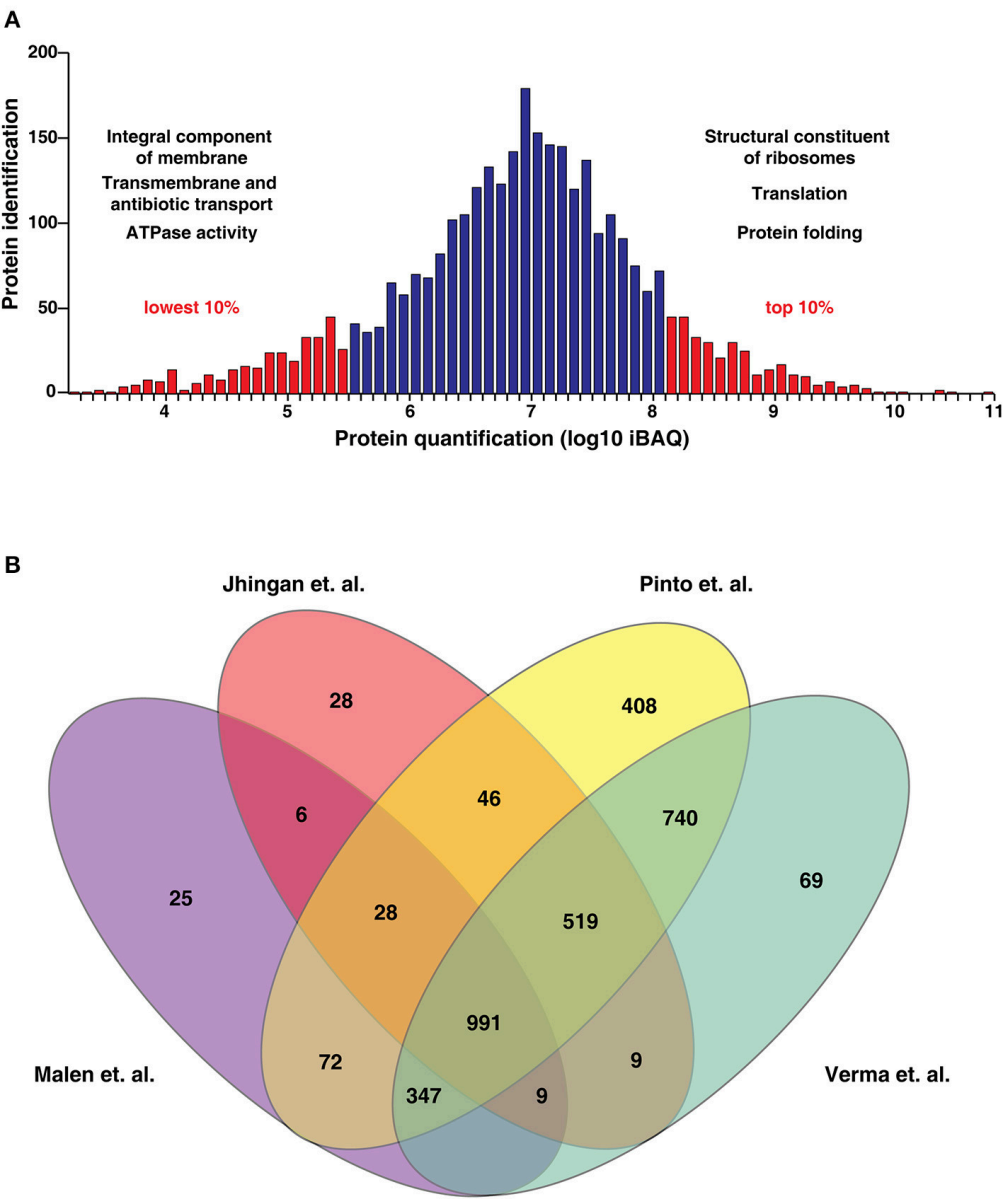
**(A)** Abundance distribution of the proteins based on the iBAQ method. (The most highly expressed 10% of proteins are dominated by proteins relating to protein synthesis, protein folding and energy metabolism. The least abundant 10% of proteins are enriched in proteins with transporter activity). **(B)** Comparison of proteins identified by Malen et al., Jhingan et al., Verma et al. and our study. We identified 408 proteins which have not been identified in previous datasets.

The identified proteins were categorized based on their molecular function, cellular localization and role in biological processes using the FunRich tool (Pathan et al., 2015; Figure 3C). Biological process-based classification revealed the enrichment of proteins involved in transcription (10.5%) and its regulation (9.7%). Further, molecular function-based classification showed ATP binding (13.7%) to be the most augmented function. Proteins such as NusA, a transcription termination/antitermination protein, TetR-family transcriptional regulator and LuxR family transcriptional regulator were among the ones found enriched in biological process-based classification. Molecular chaperones including GroES, GroEL and ATP synthase subunits were also found to be significantly enriched. Cellular component-based classification indicated that most proteins were localized to the cytoplasm (25.8%) followed by membrane. Examples of proteins localized to the cytoplasm included chromosomal replication initiator protein DnaA, Cyclopropane mycolic acid synthase 1 which play important roles in DNA replication initiation and determining cell envelope permeability, host immunomodulation, and persistence, respectively. We next looked at the abundance distribution of the proteome based on the iBAQ method (Figure 4A). The top 10% of abundant proteins were dominated by proteins involved in protein synthesis, protein folding, and energy metabolism processes. However, bottom 10% of least abundant proteins were found to be specifically enriched for proteins localized in the membrane with transporter activity. We also found enrichment of enzymes involved in cobalamin synthesis and proteins with monooxygenase activity.

### Evidence for protein-coding genes hitherto thought to be absent in H37Ra

In an earlier study reported by He et al. (2003), two-dimensional gel electrophoresis-based comparative proteome analysis of culture supernatant proteins obtained from H37Ra and H37Rv was performed to examine differential expression of proteins among the two strains. The analysis resulted in the identification of 5 proteins to be exclusively identified in H37Rv in comparison to H37Ra. These include members of the ESX protein family namely Rv2346c (esxO), Rv2347c (esxP), Rv1038c (esxJ), Rv1197 (esxK), and Rv3620c (esxW). On the basis of these findings, the authors suggested that these proteins may play an important role in virulence attenuation of H37Ra. From our analysis, we identified transcript evidence for *esxO, esxJ, esxP*, and *esxK* in the current analysis. We also observed protein expression of esxO and esxK with 5 and 11 peptides respectively (Table 1). Based on the abundance scale, these proteins range in the top 10% abundance with esxO being expressed the most. Representative MS/MS spectra are illustrated in Supplementary Figure 1A. The data obtained from this analysis refutes the findings from the previous study with evidence provided at both transcript and proteome level.

**Table 1:** List of proteins hitherto reported as absent in H37Ra.

**MRA_ID (Rv ID)**	**Gene symbol**	**Description**	**Log2 iBAQ**	**Log2 RPKM**	**H37Ra Log/H37Rv Log**	**H37Ra stationary/H37Rv stationary**
MRA_RS12470 (Rv2346c)	esxO	ESAT-6 like protein EsxO	31.07	10.43	1.3	0.9
MRA_RS06370 (Rv1197)	esxK	ESAT-6-like protein EsxK	30.84	6.07	–	–
MRA_RS12475 (Rv2347c)	esxP	ESAT-6 like protein EsxP	–	10.39	–	–
MRA_RS05510 (Rv1038c)	esxJ	ESAT-6 like protein EsxJ	–	5.87	2.1	1.7
MRA_RS19185 (Rv3620c)	esxW	ESAT-6 like protein EsxW	–	5.15	–	–
MRA_RS18460 (Rv3479)	Rv3479	Membrane protein	20.08	2.90	0.8	0.3
MRA_RS20115 (Rv3792)	aftA	Arabinofuranosyltransferase	22.30	3.98	1.2	1.0
MRA_RS12320 (Rv2319c)	Rv2319c	Universal stress protein	18.67	2.66	0.5	0.8
MRA_RS06305 (Rv1184c)	Rv1184c	Exported protein	–	2.25	0.7	1.0

We also compared our data with previously published proteomic datasets including Jhingan et al., and Malen et al. We identified 1,019 proteins common to all three data sets and 1,148 proteins unique to our data set. Further, comparison of the current proteomic data with a recent study published by our group revealed 408 proteins identified uniquely in this study (Figure 4B, Supplementary Table 5B). In the study by Malen *et al.*, which focused on the comparative analysis of membrane-associated proteins in *M. tuberculosis* H37Rv and H37Ra strains, 4 proteins were identified with 4 or more peptides in H37Rv but reported as absent in H37Ra. These include Rv3479, Rv3792, Rv2319c, and Rv1184c. Verma *et al.*, have shown lower expression of Rv3479 and Rv2319c in H37Ra when compared to H37Rv (Verma et al., 2017). We identified Rv1184c which encodes for PE-PPE domain-containing protein only at the transcript level and the orthologous counterparts of 3 proteins with evidence at both transcript and proteome level. These include Rv3479 (MRA_RS18460) which encodes for a membrane protein (Supplementary Figure 1B), arabinofuranosyltransferase (AftA) (Rv3792) involved in the biosynthesis of the mycobacterial cell wall glycan, arabinan and Rv2319c (MRA_RS12320) belonging to Universal stress protein family protein (Supplementary Figure 1C). The expression levels of the former two proteins were in the top 50% abundances whereas MRA_RS12320 was among the 10% of least abundant proteins identified (Table 1). The representative MS/MS spectra are depicted in Supplementary Figures 1A–C. In the current version of NCBI, the protein record of MRA_RS18460 (WP_003901652.1) which is the orthologous counterpart of Rv3479 is suppressed as it is no longer annotated on any genome.

We also identified a protein encoded by MRA_RS02220 (WP_003898444.1) exclusively in our dataset with log2 iBAQ value of 27.55 and log2 RPKM of 6.8. It is among the 10% most highly expressed proteins with 3 unique peptides (Supplementary Figure 1D). However, there is no entry at the protein level in the NCBI database owing to the fact that it is no longer annotated in any genome. A BLAST analysis suggested its conservation across various mycobacterial species. Our analysis thereby emphasizes the power of in-depth proteome analysis in its ability to identify coding evidence for computationally predicted genes.

### Identification of putative virulence factors

We next compared our data with the virulence-associated factors listed in Forrellad et al. (2013). Interestingly, a majority of proteins deemed as virulent factors were involved in mycolic acid and lipid synthesis. It has earlier been established that cell wall of *M. tuberculosis* plays a vital role in its virulence. It displays a wide array of complex lipids and lipoglycans on its surface, which aid the pathogen in host interaction and disease progression (Smith, 2003; Vander Beken et al., 2011). Therefore, the genes responsible for biosynthesis and transport of potential virulence factors are important from an investigative point of view and may offer new drug targets. Changes in these genes at genome, transcriptome or proteome may affect the virulence of *M. tuberculosis*. In order to characterize such changes, we mapped the mutations identified in genome analysis with the virulence-associated protein. Of the 153 proteins, genes corresponding to 6 proteins- carried mutations in H37Ra with respect to H37Rv suggesting the role of these genes in *M. tuberculosis* virulence. These include *pstA1, phoP, plcA, plcB, PE_PGRS51* and apoptosis inhibitor protein.

We further compared the list of putative virulence factors with the proteins identified in this study resulting in the identification of 139 proteins. A partial list of proteins is provided in Table 2. These proteins were further compared with the list of proteins identified in previously published studies. Altogether, our analysis provides proteomic evidence for 12 putative virulence factors identified for the first time in H37Ra proteome. These include RNA polymerase sigma factors sigE and sigL that are also members of the ECF subfamily, FAD-containing monoxygenase mymA, transporter proteins including MmpL8, lipid carrier protein (MRA_RS18785) and ABC transporter permease (MRA_RS18565) among others.

**Table 2:** Partial list of putative virulence factors identified in H37Ra.

**MRA_ID (Rv_ID)**	**Gene symbol**	**Description**	**Biological process**	**Log2 iBAQ**	**Log2 RPKM**	**H37Ra Log/H37Rv Log**	**H37Ra stationary/H37Rv stationary**
MRA_RS11925 (Rv2246)	*kasB*	3-oxoacyl-ACP synthase II	Lipids and Fatty Acid Metabolism	28.66	8.2	1.5	1.5
MRA_RS03395 (Rv0642c)	*mmaA4*	SAM-dependent methyltransferase	Mycolic acid synthesis	27.33	8.62	1	1.2
MRA_RS02465 (Rv0470c)	*pcaA*	Cyclopropane mycolic acid synthase		27.23	7.65	1.3	1.7
MRA_RS16390 (Rv3083)	*mymA* operon	FAD-containing monooxygenase MymA		17.06	3.77	ND	ND
MRA_RS16395 (Rv3084)	*lipR*	Acetylhydrolase		23.29	2.31	0.5	1.3
MRA_RS16400 (Rv3085)	*Rv3085*	Acetoin dehydrogenase		22.18	2.91	0.1	0.5
MRA_RS16405 (Rv3086)	*adhD*	Alcohol dehydrogenase		21.44	4.97	0.7	0.5
MRA_RS16410 (Rv3087)	*Rv3087*	Diacylglycerol O-acyltransferase		23.33	4.62	1	1
MRA_RS16415 (Rv3088)	*tgs4*	Diacylglycerol O-acyltransferase		22.37	4.07	0.7	0.9
MRA_RS16420 (Rv3089)	*fadD13*	Long-chain-fatty-acid–CoA ligase FadD13		22.62	4.32	0.9	0.8
MRA_RS15645 (Rv2946c)	*pks15*	Polyketide synthase	Synthesis of complex lipids	18.69	3.56	ND	ND
MRA_RS15650 (Rv2947c)	*pks1*	Polyketide synthase		22.5	3.65	ND	ND
MRA_RS20275 (Rv3823c)	*mmpL8*	transporter mmpL8	Sulfolipid synthesis	15.79	3.06	ND	ND
MRA_RS10130 (Rv1916)	*aceAb*	isocitrate lyase subunit B	Others genes related in lipid synthesis	16.84	5.81	ND	ND
MRA_RS12495 (Rv2351c)	*plcA*	Phospholipase C1		24.95	5.54	0.8	1.2
MRA_RS12490 (Rv2350c)	*plcB*	Phospholipase C2		25.86	6.24	1	1.1
MRA_RS18805 (Rv3544c)	*fadE28*	acyl-CoA dehydrogenase	Catabolism of cholesterol	20.68	3.22	ND	ND
MRA_RS18785 (Rv3540c)	*ltp2*	Lipid-transfer protein		20.44	2.27	ND	ND
MRA_RS00910 (Rv0167)	*yrbE1A*	Membrane protein	Cell wall proteins	21.46	7.88	ND	ND
MRA_RS18565 (Rv3500c)	*yrbE4B*	ABC transporter permease		20.37	4.72	ND	ND
MRA_RS07170 (Rv1348)	*irtAB*	Iron import ATP-binding/permease IrtA	Metal importer	18.06	2.78	ND	ND
MRA_RS06490 (Rv1221)	*sigE*	ECF RNA polymerase sigma factor SigE	Sigma factors	21.1	8.62	ND	ND
MRA_RS17475 (Rv3286c)	*sigF*	RNA polymerase sigma factor SigF		24.55	4.58	ND	ND
MRA_RS03875 (Rv0735)	*sigL*	RNA polymerase sigma factor SigL		20.98	3.12	ND	ND

### Identification of novel protein coding genes in H37Ra genome using genome search specific peptides (GSSPs)

The data obtained from the proteomic study was further analyzed using proteogenomics pipeline to enable refinement of genome annotation. We considered RefSeq dataset as the reference proteome for gene annotation. As described in the methods section, GSSPs identified from the proteogenomics analysis were categorized. In all, we identified 82 novel peptides corresponding to 63 novel protein-coding genes (Supplementary Table 6A). Of these novel ORFs, 10 were supported by two or more peptides whereas the remaining 53 ORFs were supported by only single peptide with acceptable spectra. Those peptides which matched to a non-coding region or were reported to be translated in a different frame were deemed as novel ORFs. Conservation of these predicted genes across mycobacterial and related species was checked using protein BLAST algorithm.

**Figure 5:**
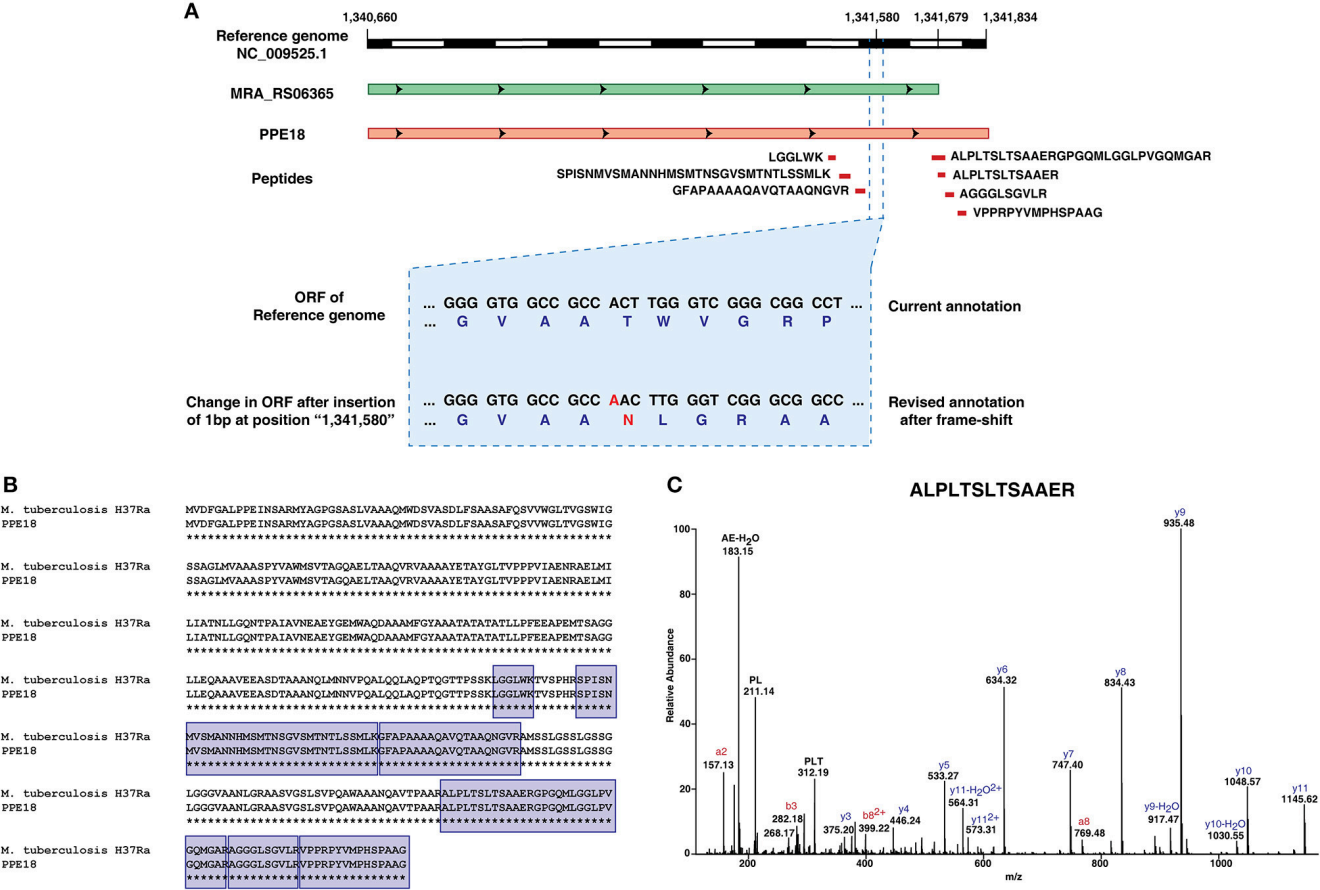
Refinement of genome annotation of *Mycobacterium tuberculosis* H37Ra genome based on transcriptomic and proteomic evidence.**(A)** Graphical representation of revised gene annotation owing to a frameshift mutation.**(B)** Orthologous evidence for the revised gene annotation.**(C)** Representative spectra of one among the 7 peptides supporting the example.

One of the interesting findings was an insertion of a base pair at position 1,341,580 in the *M. tuberculosis* H37Ra genome resulting in the frameshift in the CDS of MRA_RS06365. The finding corroborates with the insertion reported by Elghraoui *et al.*, as well. The current NCBI annotation has this gene categorized as a pseudogene. However, based on our analysis, we now provide evidence at both transcript and protein level with 7 peptides mapping uniquely to this putative pseudogene. Orthologous evidence suggests that the protein encoded by this gene is a member of the PPE family (PPE 18) and is conserved across mycobacterial species (Figure 5).

### Identification of N-terminal peptides and correction of translational start sites (TSS)

Using alternate codon database and hypothetical N-terminal tryptic peptide database, we identified events of N-terminal extension in 25 proteins (Supplementary Table 6B and 6C). Several proteins which were found to have N-terminal extension were involved in cell wall synthesis and membrane transport. Additionally, using protein N-terminal acetylation as dynamic modification, we validated protein translation start sites of 332 proteins reported in H37Ra reference database (May 2016). These include high abundant proteins from ESX secretion system of mycobacteria such as ESX-1 secretion-associated protein EspB, EspF, and EspJ. We also identified 3 incidences of ICDSs in H37Ra genome of which, one was attributed to a potential sequencing error and the other two to stitching errors.

## Discussion

Integrated analyses of multi-omics data have proven to be powerful tools to understand complex biological questions (Prasad et al., 2017). Whole genome sequencing of *M. tuberculosis* H37Ra carried out in this study confirmed 39 SNVs that were reported in earlier studies. Interestingly, we identified 46% (18/39) of these mutations in the genes encoding PE-PGRS family proteins (Supplementary Table 3A). The members of the PE-PGRS family have been shown to play a role in evasion of host immune responses, possibly via antigenic variations (Sampson, 2011). Further, we also confirmed stop gain mutations in two H37Ra genes namely phosphate ABC transporter permease *pstA* and oxidoreductase. *pstA* is involved in active transport of inorganic phosphate across the membrane (Braibant et al., 2000), oxidoreductase, on the other hand, is involved in cellular respiration (Sassetti et al., 2003). Of the 14 high confidence H37Ra-specific variants previously reported by Elghraoui group, we identified mutations in *phoP* (S219L) and *espK* (I228M) genes. PhoP is part of two-component regulatory system PhoP/PhoQ. It is involved in transcriptional regulation of several virulence-associated genes and is also required for intracellular growth of *M. tuberculosis* (Pérez et al., 2001). Mutations in the *phoP* gene have been implicated in reduced survival and persistence of mycobacteria inside the host cell. PhoP controls the biosynthesis of sulfolipid (SL) and di- and polyacyltrehaloses (DAT and PAT) (Gonzalo Asensio et al., 2006; Walters et al., 2006). The mutation S219L in *phoP* gene affects the OmpR/PhoB-type DNA-binding which spans from 148 to 245 amino acids suggesting the role of this mutation in virulence attenuation. We identified the expression of *phoP* at both transcript and proteome level at higher abundance compared to the other proteins identified in this study.

Comparison of the In-Dels identified in our analysis with the structural variations reported by Elghraoui and Zheng *et al.*, resulted in the identification of 14 insertions of which 8 led to frameshift mutations, 5 upstream variants and one inframe insertion (Supplementary Table 3B). We confirmed 9 deletions to be common among all studies, 3 of these being large deletions including *nrdH*-redoxin, *IS6110* transposase and IS3 family transposase. NrdH is involved in electron transfer system for ribonucleotide reductase system. Phenotype profiling studies in *M. tuberculosis* has identified this protein to be essential for growth and cholesterol metabolism (Griffin et al., 2011). Transposable elements such as *IS6110* can affect an organism's fitness through the insertional inactivation of genes (Yesilkaya et al., 2005). Carlos and groups have shown that *IS6110* copy located within the promoter region of *phoP* affects the expression of several virulence-associated genes (Soto et al., 2004). Large deletions identified in *IS6110* transposase need to be further investigated to study its role in virulence of *M. tuberculosis*. Among the genes identified with upstream deletion, we identified polyketide beta-ketoacyl synthase *pks3*, a gene well known to be involved in the cell wall lipid biosynthesis (Dubey et al., 2002).

### Protein coding evidence for genes supposed to be “absent” in H37Ra

Our integrated analysis provides evidence of translation for 5 genes that were previously thought to be absent in H37Ra. Additionally, evidence at the transcript level was obtained for 9 genes inclusive of the above mentioned 5 genes. These include members of the esx5 cluster namely *esxO* and *esxK*, as well as an enzyme required for arabinan synthesis (*aftA*) and a member of the universal stress family proteins. The findings from this study refute the reports published earlier with evidence provided at both transcript and proteome level. Further, a recently published study by our group could also quantify these proteins. Interestingly, the abundance of these apparently missing proteins did not vary significantly in both H37Rv and H37Ra strains (Table 1). For example, esxO recently shown to be involved in promoting intracellular persistence and host cell invasion upon ectopic expression in *M. smegmatis* (Mohanty et al., 2016), was expressed at similar levels in both log and stationary phase of H37Ra in comparison to H37Rv. A study on esxO also reports induction of autophagy and genomic instability in infected cells due to increase in host oxidative stress response. This suggests that there could be other mechanisms probably involving post-translation modifications that may likely play a role in modulating mycobacterial pathogenesis. Similarly, *aftA* encoding arabinofuranosyltransferase and *Rv1184c* was expressed almost equally in both the strains. Interestingly, *esxJ* which was identified only at the transcript level in our study was expressed more that 2-fold in the log phase of H37Ra in comparison to H37Rv.

### Proteomic evidence for putative virulence factors identified in H37Ra

Several investigations have been carried out for the identification of genes essential for mycobacterial virulence to understand the molecular basis of their virulence and persistence. Most of these genes encode for enzymes involved in lipid synthesis pathway, cell surface proteins encoding genes, regulators of metabolism and bacterial secretion system (Smith, 2003, Vander Beken et al., 2011). Genomic investigation of H37Ra from the current analysis revealed non-synonymous variations in six virulence-associated genes including membrane-associated phospholipase C1 and phospholipase C2 genes. Studies have shown that phospholipase C mutants in *M. tuberculosis* are attenuated in the late phase of the infection emphasizing the importance of phospholipases C in the virulence of the tubercle bacillus (Raynaud et al., 2002). Additionally, we provide evidence of expression of these genes at both transcript and proteome level, emphasizing expression of possibly non-functional phospholipases C1 and C2.

Further, comparative analysis of the putative virulence factors reported by Forrellad et al., with proteins identified uniquely in this study resulted in the identification of protein products of 12 putative virulence-associated genes. These proteins are known to be involved in the survival of mycobacterium inside the adverse microenvironment of host macrophage (Table 2) and include proteins belonging to the RNA polymerase sigma factor family namely, SigE and SigL, FAD-containing monoxygenase MymA, transporter proteins including mmpL8 among others. Interestingly, our study provides transcriptomic and proteomic evidence for all the seven genes belonging to the *mymA* operon. The operon in *M. tuberculosis* encode for proteins required for the proper ultrastructure of the cell envelope (Singh et al., 2005). Although *Rv3083* or *mymA* has been identified in other mycobacterial species, our study provides the first proteomic evidence for its expression in *M. tuberculosis* H37Ra. This gene is known to have a role in modifying mycolic acid structure on the cell envelope of the mycobacteria thus helping them survive the acidic environment inside the macrophages (Saraav et al., 2017). The expression level of *mymA* in our data is comparatively lower than the other genes comprising this operon. It is therefore likely to have been missed in earlier studies owing to its low expression levels. Comparison with the expression profile from our previous study revealed that the first few genes of the *mymA* operon (*Rv3084* and *Rv3085*) were expressed at a lower level in H37Ra as compared to H37Rv (Verma et al., 2017). Additionally, Verma *et al.*, have identified 35 putative virulence factors of which, 13 and 22 proteins were found to be upregulated and downregulated, respectively in H37Ra with respect to H37Rv (Fold change ≥ 2). Among these, HspX was found to be more than two-fold downregulated in H37Ra stationary phase. This protein is known to possess chaperone activity with a significant ability to suppress the autolysis of *M. tuberculosis* and slow down the growth rate of bacteria during macrophage infection providing a survival advantage.

### Refinement of genome annotation through proteogenomics analysis

Using an integrated proteogenomic workflow, we report the identification of several novel peptides that enable correction of errors in genome assembly and annotation of gene models. Comparison of peptides corresponding to novel genes with peptides identified in Verma *et al.*, revealed identification of 2 novel genes in H37Ra, which have been missed in the earlier genome annotation. Of this, one was found to be similar to *PPE18* and the other to *Rv1586c*. Both these genes are expressed to a similar extent in both H37Ra and H37Rv. PPE18 is known to be required by the tuberculosis bacilli for its intracellular survival in the host and Rv1586c is known to play a role in integrating phiRv1, one among the 2 pro-phages common in the MTBC genomes, to its own genome (Fan et al., 2016). Thus, both of them are virulence associated factors in the virulent strain. Their identification in the avirulent strain further warrants the need for analysis that will ascertain the role of the currently known virulent factors.

Accurate annotation of the translational start sites (TSS) using gene prediction tools still remains a major challenge. It has been estimated that 60% of the assigned TSS are erroneous (Nielsen and Krogh, 2005). The genome of H37Ra shows high similarity with that of H37Rv because they originated from a common parental strain (Jhingan et al., 2016). Approximately 60, 33, and 5% of the genes in *M. tuberculosis* are known to use ATG, GTG, and TTG as start codons, respectively (Kelkar et al., 2011). Although the initiator methionine is known to undergo formylation (fMet) in prokaryotes, N-terminal acetylation of proteins has also been observed in them, albeit at a lower rate compared to eukaryotes (Jones and O'Connor, 2011). Identification of these modified peptide sequences using mass spectrometry can be exploited to validate protein TSS (Prasad et al., 2017).

*In silico* studies have shown that bacterial genomes may comprise of undetected frameshift mutations (Elghraoui et al., 2017). Unrecognized frameshifts, stop codons in the frame and sequencing errors lead to interrupted coding sequences (ICDS) that can seriously affect the subsequent genome analysis. ICDS may be present in any gene of known or unknown function. For restoring the correct reading frame, several bacterial species have adopted the mechanisms of bypassing frameshifts. However, such incidences are rare. These sequences may be truly present in the organism and missed during sequencing or may arise from misannotation due to sequencing errors. One of the ICDSs identified in this study (NC_009525.1: 2,247,871-2,248,081), was found to be supported by 5 PSMs in proteomics analysis, where the sequence of known gene shifts open reading frames from frame 2 to frame 1. The resulting protein sequence mapped to a hypothetical protein (WP_057368341.1) of H37Rv strain.

## Conclusions

In this study, we successfully demonstrated the utility of multi-omics approaches for the validation and correction of genomic data. Although a recent study suggested sequencing errors in the genome of H37Ra, no evidence was provided at the transcriptomic and proteomic levels. In the current study, we confirmed the identification of the genomic differences reported in previous studies. Furthermore, our study also provides evidence of the mutation spectrum in the promoter regions of certain virulence-associated genes. One of the important findings of this study include evidence of transcription and translation of genes that were previously reported as absent in H37Ra. These include genes that have been implicated in the mechanism of virulence and pathogen persistence in the host. Using an integrated proteogenomics pipeline, we also provide evidence of translation of several novel ORFs that have been annotated in other strains of *M. tuberculosis* including the virulent strain H37Rv but were missing in the genome annotation of H37Ra. The data generated from this study will pave the way for future studies on understanding the mechanism of virulence attenuation in H37Ra.

## Author contributions

SP, SG, and TP contributed to conception and design of the study. SG provided the samples for the study. SP, RV, RR, and SK processed the samples. SP acquired mass spectrometry data. SP, RV, JA, OC, AP, SK, and YS analyzed the data. SP, JA, RV, OC, and AP prepared tables and figures. SP, RV, OC, SK, and YS carried out the literature search and wrote sections of the manuscript. All authors contributed to manuscript revision, read and approved the submitted version.

### Conflict of interest statement

The authors declare that the research was conducted in the absence of any commercial or financial relationships that could be construed as a potential conflict of interest.

## Supplementary material

Supplementary Figure 1 Representative MS/MS spectra of the proteins identified uniquely in our study and earlier reported to be absent in H37Ra.

Supplementary Table 1 **(A)** List of mutations identified in H37Ra when data was aligned with H37Ra reference genome (Zheng et al.). **(B)** List of INDELS identified in H37Ra when data was aligned with H37Ra reference genome (Zheng et al.). **(C)** List of mutations identified in H37Ra when data was aligned with H37Ra reference genome (Elghraoui et al.). **(D)** List of INDELS identified in H37Ra when data was aligned with H37Ra reference genome (Elghraoui et al.).

Supplementary Table 2 **(A)** List of mutations identified in H37Ra when data was aligned with H37Rv reference genome. **(B)** List of INDELS identified in H37Ra when data was aligned with H37Rv reference genome. **(C)** List of mutations in the promoter regions of 7 genes.

Supplementary Table 3 **(A)** List of mutations common in our study, Zheng et al. and Elghraoui et al. **(B)** List of INDELS common in our study, Zheng et al. and Elghraoui et al.

Supplementary Table 4 List of transcripts identified in this study.

Supplementary Table 5 **(A)** List of proteins and peptides identified by LC-MS/MS analysis *Mycobacterium tuberculosis* H37Ra strain. **(B)** Comparative analysis of the list of proteins identified in previous published data and data from this study

Supplementary Table 6 A summary of results from proteogenomics analysis. **(A)** Novel genes. **(B)** Protein extensions. **(C)** Alternate start site.

## Abbreviations

BLAST: Basic Local Alignment Search Tool
PSM: Peptide Spectrum Match
FDR: False Discovery Rate
ICDS: Interrupted Coding Sequences
GSSP: Genome Search Specific Peptides
iBAQ: intensity Based Absolute Quantification
NCBI: National Centre for Biotechnology Information.
